# Management of the Oral Health of Children During the COVID-19 Pandemic in Poland

**DOI:** 10.3389/fpubh.2021.635081

**Published:** 2021-07-29

**Authors:** Aneta Olszewska, Elzbieta Paszynska, Magdalena Roszak, Agata Czajka-Jakubowska

**Affiliations:** ^1^Department of Developmental Facial Malformation, Poznan University of Medical Sciences, Poznań, Poland; ^2^Department of Integrated Dentistry, Community Dentistry Section, Poznan University of Medical Sciences, Poznań, Poland; ^3^Department of Computer Science and Statistics, Poznan University of Medical Sciences, Poznań, Poland; ^4^Department of Temporomandibular Joint Disorders, Poznan University of Medical Sciences, Poznań, Poland

**Keywords:** oral health, children, COVID-19 pandemic, dentistry, protocols

## Abstract

Managing the oral health of children during the time of a health emergency linked to the current COVID-19 pandemic presents specific problems. A high number of non-specific effective infection control protocols are available in dental settings. It is of fundamental importance to implement specific protocols relating to those clinical situations that normally do not represent an emergency but which now fall into that category. The aim of this study was the comparison of data obtained from the Regional National Health Fund (NFZ) relating to the number and the type of procedures in the oral health management of children aged 0–18 years from the Wielkopolska region, with the months of March and April of 2019 being compared with those of the, respective, pandemic period of 2020. The results showed statistical differences in the number of performed procedures when comparing 2019 and 2020; especially in April (*n* = 53,077 in 2019 but only *n* = 2,287 in 2020), when lockdown restrictions reached their highest level and when only 30% of the dental clinics for children were open for patients in the Wielkopolska region of Poland. Regarding surgical cases, there were no differences in percentage frequency between April 2019 and 2020 in terms of extractions. However, an increase was observed in abscess incisions (3.5–17.8%) and surgical dressings (1.5–10.07%). There was a decrease in the total number of performed conservative dentistry procedures in April 2020, but temporary fillings in primary and permanent teeth showed a prominent increase: from 6.4% in 2019 to 19.3% in 2020; and 5.8–11.4%, respectively. Pulp treatment and mucosal lesions therapy fall into the dental emergency category during this COVID-19 pandemic. These cases have shown an increase from 3.2% in 2019 to 12.8% in 2020 for pulp treatment, and from 2.3 to 4.3% for the treatment of oral mucosal lesions. As suspected, after the lockdown was implemented, the number of pediatric dental cases were low. Moreover, the analysis revealed differences in the profile of clinical situations that represented the emergency cases and the pandemic treatment protocols. Future implications suggest that dental prophylactic procedures be included in pandemic protocols with even dental services being limited to a form of urgent treatment. New approaches and treatment models should be implemented in the control of the infectious spread of the disease in the management of the oral health of children in this pandemic period.

## Introduction

During the new SARS-CoV-2 virus pandemic, countries implemented different measures for the delivery of dental care for adult and pediatric patients (1). Many dental procedures have been associated with the transmission of SARS-Cov-2 via droplets and splatter from blood and saliva through direct and indirect contact. Direct transmission occurs through respiratory droplets by coughing or sneezing, or in the course of dental procedures between the dentist, the assistant, and the patients. The risk of indirect transmission can occur when a patient or the dental team are in contact with surfaces contaminated with the virus (dental chair, spittoons, floor, etc.). Some procedures, such as using a high-speed turbine, ultrasonic scaler and spray gun, produce a large amount of aerosol, which can remain suspended in the air for long periods of time, increasing the risk of transmission (2, 3).

Because children can be asymptomatic or present with non-specific symptoms, it has been recommended that all pediatric patients and their parents/caregivers should be considered as possible carriers of SARS-CoV-2. This potentially puts pediatric dentists at a high risk of infection (4–7). The management of pediatric patients can also be influenced by behavioral problems connected with greater dental anxiety due to the use of personal protective equipment (mask, goggles, face shield) which makes conversation and adaptation to the dental environment very difficult (8).

During the COVID-19 epidemic (March/April 2020), according to the national recommendations for medical specialties, the routine dental treatment for children in Poland was suspended and limited to emergency cases only. It was reported that only 30% of the dental clinics for children were open for patients in the Wielkopolska region of Poland.

Therefore, the objective of this study was to compare a number dental cases performed in both children and adolescents with national insurance before (March and April 2019 y) and during the actual phase of COVID-19 (March and April 2020 y).

## Materials and Methods

Approval by the Ethics Committee has been revised by the project protocol at the Poznan University of Medical Sciences (9/06/2020). The analysis was based on data from dental offices in Wielkopolska, which have signed a contract for medical services agreed to be performed in dental care in pediatric dentistry. The data collection was obtained from the National Health Fund (NFZ) under its administrative agreement as well as freedom in access to information guaranteed by national law regulations. The patient status was obviously kept anonymous. One of the examiners involved in data extraction holds the function of a qualified consultant in pediatric dentistry (A.O.). A comparison of the dental service from March/April 2019 vs. March/April 2020 was conducted. The above-mentioned period of time was chosen as a blow up of the first SARS-CoV-2 pandemic lockdown in relation to community activities. Dental service procedures for surgery (S) and conservative dentistry (CD) were divided into the following categories: tooth extraction: single-root (category S), tooth extraction: multiple-root (S), surgical dressing (S), abscess incision (S) and temporary filling of secondary teeth (category CD), temporary filling of primary teeth (CD), restoration of primary teeth (CD), restoration of secondary teeth (CD), post-trauma reconstruction of tooth (CD), trepanation (CD), devitalization (CD), and other procedures (CD). It is noteworthy that during the severe phase of the SARS-CoV-2 pandemic, regular prosthodontic, orthodontic, and periodontal procedures were suspended and they were not considered as marginal in the analyzed period of time.

In 2019, 97 dental clinics in Wielkopolska provided dental services for children and adolescents up to the age of 18. In 2020, this number increased to 99 offices that were dedicated to treating children.

The dental offices are required to submit a report each month for counter-work. The work done during the pandemic period did not release these dental offices from the obligation to submit these reports.

Only correctly filled forms of the reports were accepted in the study and these served as a sources of data. In this study, we have not analyzed data according to age, sex, location of dental practice, specialty of dental practitioners, or any level subject to satisfaction with the provided dental services.

According to the information provided by the Central Statistical Office (GUS), the population of children and adolescents (up to 18 years of age) in Wielkopolska in 2019 stood at 744,228.

### Statistical Analysis

The data are presented as absolute numbers and/or percentages, as appropriate. The categorical data (2 × 2 or larger contingency tables) were analyzed using the Chi-squared test for independence and/or as the difference between the two proportions. All results were considered significant at *p* < 0.05. All statistical analyses were performed with InStat 3.06 (GraphPad Inc.) and Statistica 13.0 (StatSoft Inc.).

## Results

The results showed statistical differences in the number of performed procedures when comparing 2019 and 2020; especially in April (*n* = 53,077 in 2019 but only *n* = 2,287 in 2020), when lockdown restrictions reached their highest level and when only 30% of dental clinics for children were open for patients in the Wielkopolska region of Poland (Table 1).

**Table 1:** The statistical comparison in the number of dental service procedures for surgery and conservative dentistry (primary and secondary dentition) performed in March and April 2019 and 2020 y.

	**2019**	**2019**	**2020**	**2020**	Differences between months ^*^
	**March**	**April**	**March**	**April**	**Multiple comparisons (** ***p*** **-value)**
**Dental surgery procedures**	***n*** **= 1,440**	***n*** **= 1,283**	***n*** **= 564**	***n*** **= 112**	
Tooth extraction: single-root	556	487	204	31	April 19 vs. April 20 (*p =* 0.031)
Tooth extraction: multi-root	829	731	326	49	April 19 vs. April 20 (*p =* 0.007) March 20 vs. April 20 (*p =* 0.007)
Surgical dressing	28	19	17	12	April 19 vs. April 20 (*p* < 0.001) March 20 vs. April 20 (*p =* 0.001)
Abscess incision	27	46	17	20	April 19 vs. April 20 (*p* < 0.001) March 19 vs. April 19 (*p =* 0.009) March 20 vs. April 20 (*p* < 0.001)
**Conservative dentistry procedures**	***n*** **= 8,127**	***n*** **= 7,517**	***n*** **= 3,501**	***n*** **= 352**	Multiple comparisons (***p***-value) ^**^
Temporary filling of secondary teeth	462	433	199	40	April 19 vs. April 20 (*p* < 0.001) March 20 vs. April 20 (*p* < 0.001)
Temporary filling of primary teeth	525	480	205	68	April 19 vs. April 20 (*p* < 0.001) March 20 vs. April 20 (*p* < 0.001)
Restoration primary teeth	4,074	3,847	1,709	12ggz6	April 19 vs. April 20 (*p* < 0.001) March 20 vs. April 20 (*p* < 0.001)
Restoration of secondary teeth	1,999	1,840	915	50	April 19 vs. April 20 (*p* < 0.001) March 20 vs. April 20 (*p* < 0.001)
Post-trauma reconstruction of tooth	70	62	36	2	ns
Trepanation	265	241	100	45	April 19 vs. April 20 (*p* < 0.001) March 20 vs. April 20 (*p* < 0.001)
Devitalization	236	169	99	15	April 19 vs. April 20 (*p =* 0.016) March 19 vs. April 19 (*p =* 0.048)
Other procedures	496	445	238	6	April 19 vs. April 20 (*p* < 0.001) March 20 vs. April 20 (*p* < 0.001)

*n, number of total procedures;*

* *statistical significance difference using the χ^2^-test p < 0.05*;

** *statistical significance difference using the χ^2^-test except post-trauma reconstruction of toot; non-significance p > 0.05 (ns)*.

**Figure 1:**
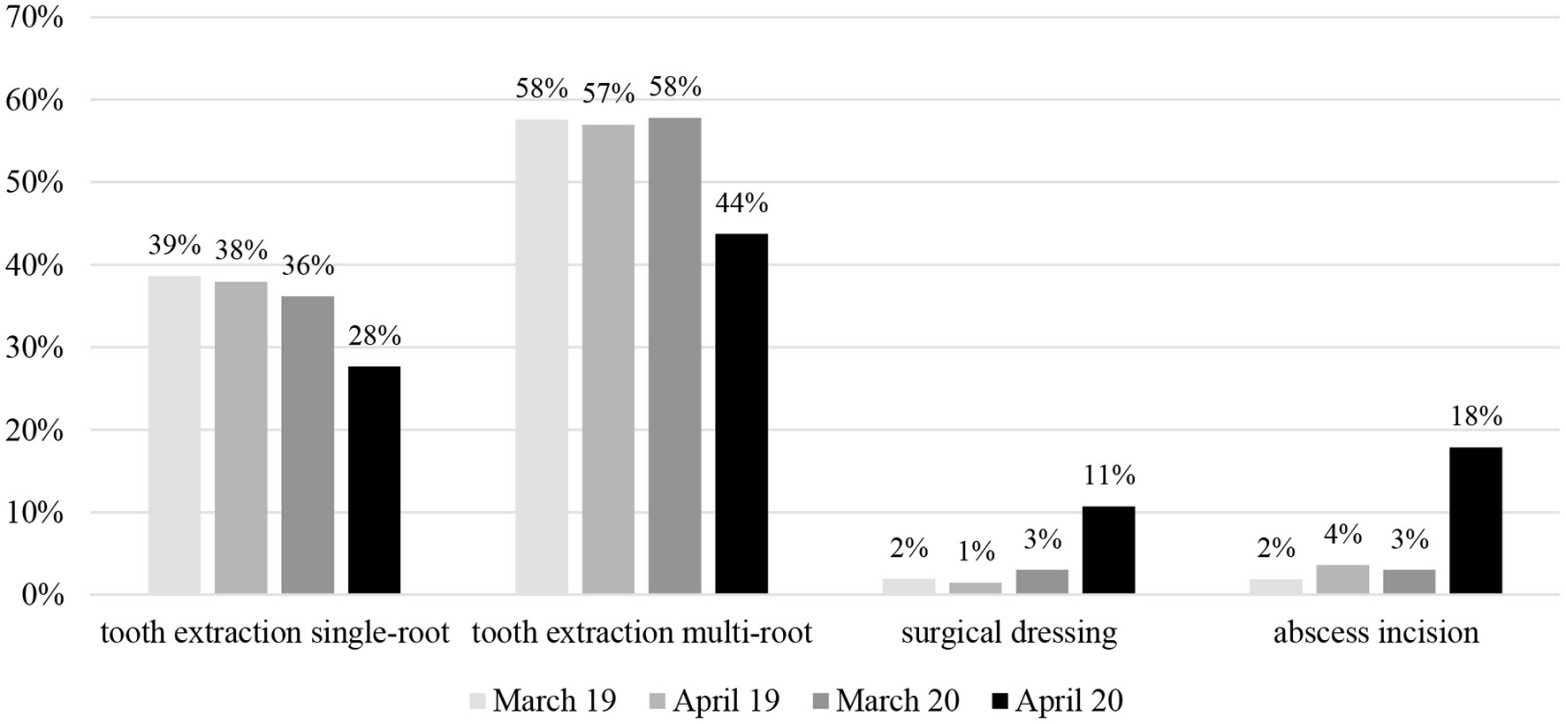
The Statistical comparison showing the percentage of the dental service procedures for surgical procedures (primary and secondary dentition) conducted in March and April 2019 and 2020 y.

**Figure 2:**
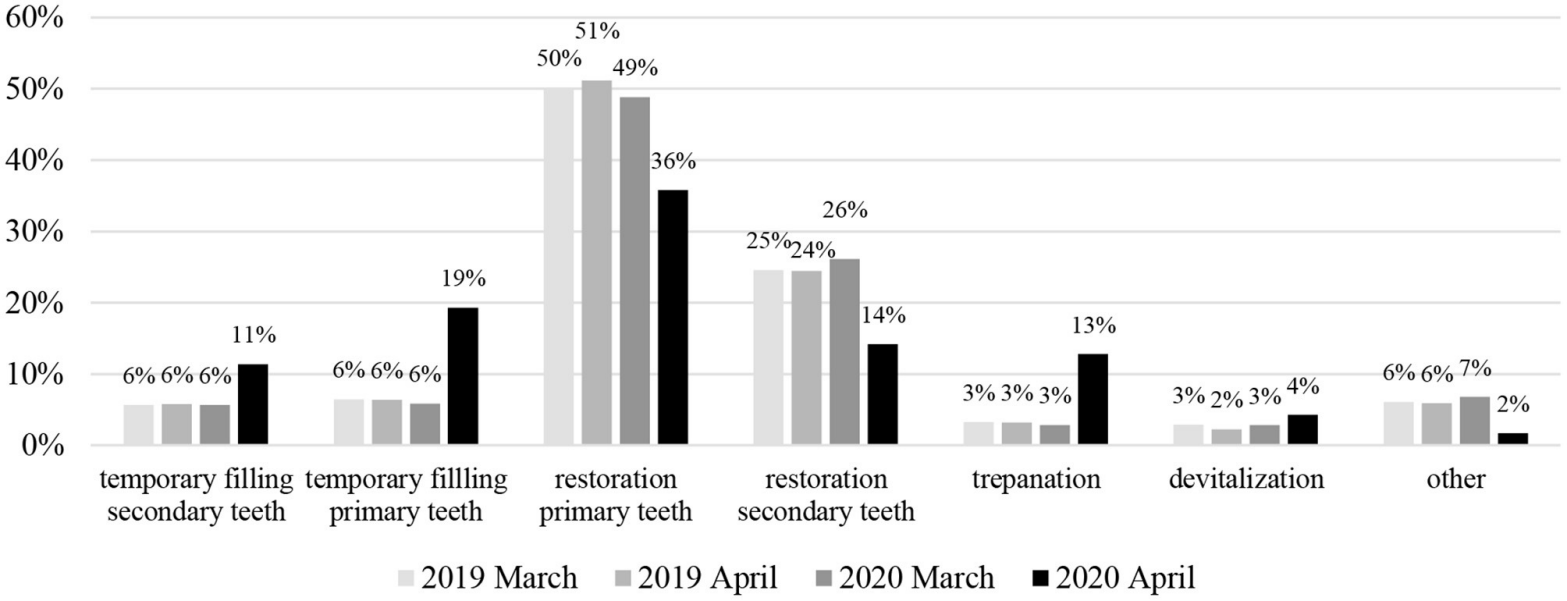
The Statistical comparison showing the percentage of dental service procedures for conservative dentistry (primary and secondary dentition) performed in March and April 2019 and 2020 y.

The statistical analysis revealed changes in the profiles of clinical situations that represented emergency cases and pandemic treatment protocols (Figures 1, 2).

Regarding the surgical cases, there were no differences in percentage frequency between April 2019 and 2020, in terms of extractions.

No correlation was seen between the month/year and the number of extractions in both single-rooted and multi-rooted teeth, and the distribution of services was similar in all the periods analyzed, namely March 2019, April 2019, March 2020, and April 2020 (*p* = 0.9186), despite the fact that fewer surgical procedures were performed in 2020.

However, an increase was observed in abscess incisions (3.5–17.8%) and surgical dressings (1.5–10.07%) (Table 1, Figure 1).

There was a decrease in the total number of performed conservative dentistry procedures in April 2020, but temporary fillings in primary and permanent teeth showed a prominent increase: from 6.4% in 2019 to 19.3% in 2020; and 5.8–11.4%, respectively.

For deciduous and permanent teeth, in March 2019, April 2019, and March 2020, the distribution of the number of therapeutic dressings and fillings was similar and there were no statistical differences. Only April 2020 was different, where the number of fillings decreased in relation to an increased number of dressings (*p* < 0.0001) (Table 1).

Taking into consideration the relationship between the time period (month/year) and the performance of the trepanation/devitalization procedures, there was a different distribution of trepanation/devitalization in the month of April 2020 when compared to the other months analyzed (*p* < 0.002): more trepanation was performed in relation to devitalization, with the ratio changing to 75/25% (previously it was 52.9/47.1%; 58.8/41.2%; 50.3/49.7%) (Figure 2).

Pulp treatment and mucosal lesions therapy fall into the dental emergency category in this COVID-19 pandemic. These cases showed an increase from 3.2% in 2019 to 12.8% in 2020 for pulp treatment and from 2.3 to 4.3% for the treatment of oral mucosal lesions.

## Discussion

As suspected after the lockdown was implemented, the number of pediatric dental cases were low. Moreover, the analysis revealed differences in the profile of clinical situations that represented emergency cases and pandemic treatment protocols.

During the first phase of the COVID-19 pandemic, restricting dental interventions to urgent and emergency cases showed that pediatric dentists have to reassess the benefits of treatment and the risks for the patient associated with potential infection. In this context, good cooperation with the parents/guardians of the child with regard to assessing the health condition and the observed symptoms as well as the measures that can be taken if a visit to the office of the dentist is not essential, as minor issues can be managed at home, makes it possible for pediatric dentists to effectively diagnose and treat patients through the use of “teledentistry.”

Based on information from the literature, clinical conditions that are not classified as urgent dental interventions and can be managed by parents/guardians at home include, among others, loss of temporary fillings in deciduous and permanent teeth which is not accompanied by pain; delayed exfoliation of deciduous teeth; and pericoronitis around the erupting permanent molars (2).

The new experience of the COVID-19 pandemic shows how important the roles played by the development of information and communication technology and the ability to remotely solve dental problems that do not require a visit to a dental office are in doctor–patient communication (9).

Considering the effects of the pandemic-related restrictions on the provision of dental services, it seems that when the pandemic is over it will be necessary to adapt to preventive and therapeutic procedures in pediatric dentistry based on the new challenges and greater treatment needs. The effects of abandoning preventive treatment in children is likely to result in a rapid increase in the need for therapeutic intervention, and reinstating regular preventive visits ought to be the first stage in the post-pandemic resumption of operations of pediatric dental offices.

During the SARS-CoV-2 pandemic, the new epidemic situation requires the implementation of special protocols and precautions, in line with the recommendations and the guidelines issued by local and global health authorities.

The safety and health of children is always a priority, especially in the dynamic conditions of the pandemic. The concerns of parents on the potential risks associated with the visit of their child to the office of the dentist can often be the reason for refraining from making an appointment, which in turn may lead to the worsening of the original problem accompanied by complications and severe pain (2).

A child who comes to the dentist with severe pain, especially during the pandemic, is at a high risk of internalizing the unpleasant sensations associated with a dental visit. In the future, this may lead to anxiety that may make it impossible for them to cooperate during dental treatment, requiring sedation via inhalation or general anesthesia.

The role of a pediatric dentist is to educate the parents/guardians of the children about the safety of the procedures performed at the office of the dentist during this particularly difficult pandemic period, while at the same time making them aware of the risks associated with abandoning or delaying dental interventions (10).

It is noteworthy that regular prosthodontic, periodontal, and orthodontic procedures were suspended due to risk of bioaerosol contamination risk at the dental clinics and, hence they were not performed. The reason for this decision lies in significant exposure to infection to pathogens from the mouth and respiratory tract of the patients (11). When working with rotary burs that require water cooling, bioaerosol is formed and the water drops act as carriers of infectious microorganisms. In 2020, the Ministry of Health completed the group of the highest risk factors for infection with biological material by including the Sars-CoV-2 virus transmitted by droplets on the list. The dynamic COVID-19 pandemic forced changes in the organization of work in dental offices based on infection prevention protocols. It is assumed that the longer bioareosol treatment, the higher the risk of COVID-19 infection in the operating areas in dental clinics. Therefore, it is believed that exposure is one of the highest cause of infection among all medical professions. Additionally, if the patient is a child who cries or screams, the risk increases significantly due to the speed of the bioaerosol trajectory. The present COVID-19 time has forced the implementation of safe work procedures with the recommendation to use effective personal protective equipment. For example, use of disposable PPE kits, face shields, masks, caps, protective materials in clothing resistant to fluids, and moisture with hermetic eye protection are recommended for dental personnel.

The recommendations of dental teams with regard to the dental visits of children during the pandemic include the presence of only one parent/caregiver in the office of the dentist; the use of oxidizing rinses (H_2_O_2_) or chlorhexidine (CHX) before examining the mouth of the child; isolating the treatment area with rubberdam; four-handed work; and the use of high-power suction systems.

The procedure should include all the necessary interventions aimed at relieving the pain of the patient at the given moment, as well as those aimed at preventing the development of other potential ailments; therefore, it is recommended that a dental clearance examination of the mouth of the child should be performed during the same visit (12).

Similar approaches to dental patients may include rinsing with commercial mouthrinses to reduce oral viremia from saliva and oral mucosa. There are several active ingredients of mouthwash that have been tested for their anticovid efficacy. These include chlorhexidine gluconate, cetylpyridine chloride, povidone iodine, hydrogen peroxide, cyclodextrin, and essential oils (13). Other reports confirm the effective protection against the transmission of SARS-CoV-2 due to the content of zinc and stannous fluoride in mouthrinses and toothpastes. In all cases, it is indicated that the exposure to the oral cavity of the patient and the concentration of an active ingredient may have a crucial role in reducing virus transmission (14).

Work safety against the spread of infectious bioaerosols has significantly increased the amount of medical wastes. Currently these materials are not biodegradable, not recyclable, and require radical disposal by complete incineration. This form of waste reduction is not ecological and burdens our environment with harmful gas compounds. Therefore, there is a need to improve protective materials, both for the safety of the natural environment and to provide better protection for medical personnel as well. The search for new solutions will also ensure a sense of security and verify the readiness of stationary dental care. Moreover, it is important for the academic education in all medical professions, which is currently a great challenge (15–19).

To assume, the end of the pandemic will have to mean the beginning of new methods of management in pediatric dentistry. Intelligent technology systems would prefer a remote dentist–patient communication as a standard tool for projecting oral health education in children, especially in the school age. Virtual contact as teleprophylaxis or dental tele-advice will be helpful in strengthening healthy habits and motivating children to follow hygienic and dietary recommendations. As recommended, minimally invasive procedures should be employed, but also strenuous efforts should be made to promote preventive measures.

The pandemic has exacerbated inequalities in health care access. Changing the financing of services for children and adolescents and shifting funds from the model of reimbursement of reconstructive treatments to prophylaxis are currently the expected models in dental care.

## Conclusions

During the first phase of the COVID-19 pandemic, restricting dental interventions to urgent and emergency cases showed that pediatric dentists had to reassess the benefits of treatment and the risks for the patients associated with potential infection.

The analysis revealed changes in the profile of clinical situations that represented emergency cases and pandemic treatment protocols. Future implications suggest that dental prophylactic procedures be included in pandemic protocols with even dental services being limited to a form of urgent treatment.

## Data Availability Statement

The original contributions presented in the study are included in the article/supplementary material, further inquiries can be directed to the corresponding author/s.

## Author Contributions

MR is responsible for statistics analyzes. AC-J for ideas and revisions of the research. All authors contributed to the article and approved the submitted version.

## Conflict of Interest

The authors declare that the research was conducted in the absence of any commercial or financial relationships that could be construed as a potential conflict of interest.

## Publisher's Note

All claims expressed in this article are solely those of the authors and do not necessarily represent those of their affiliated organizations, or those of the publisher, the editors and the reviewers. Any product that may be evaluated in this article, or claim that may be made by its manufacturer, is not guaranteed or endorsed by the publisher.
